# Life-Threatening Macrophage Activation Syndrome in Pregnancy: First Manifestation of SLE Induced by Parvovirus B19

**DOI:** 10.3390/ijms26115406

**Published:** 2025-06-04

**Authors:** Aleksandra Plavsic, Rada Miskovic, Dragana Jovanovic, Uros Karic, Zikica Jovicic, Sara Radovic, Ana Drazic, Aleksandra Dasic, Snezana Arandjelovic

**Affiliations:** 1 Clinic for Allergy and Immunology, University Clinical Centre of Serbia, 11000 Belgrade, Serbia; rada.miskovic@med.bg.ac.rs (R.M.); dragana.r.jovanovic@med.bg.ac.rs (D.J.); zikica1363@gmail.com (Z.J.); drazic998@gmail.com (A.D.); aleksandra.dasic777@gmail.com (A.D.); snezana.arandjelovic@med.bg.ac.rs (S.A.); 2 Faculty of Medicine, University of Belgrade, 11000 Belgrade, Serbia; 3 Clinic for Infectious and Tropical Diseases, University Clinical Centre of Serbia, 11000 Belgrade, Serbia

**Keywords:** hemophagocytic lymphohistiocytosis (HLH), P (MAS), systemic lupus erythematosus (SLE), parvovirus B19 (P19V), pregnancy

## Abstract

Macrophage activation syndrome (MAS) is a complex, life-threatening, hyperinflammatory condition occurring as a form of hemophagocytic lymphohistiocytosis (HLH), commonly associated with several autoimmune and autoinflammatory diseases, and certain infections such as Parvovirus B19 (P19V). The onset of systemic lupus erythematosus (SLE) presenting as MAS during pregnancy is uncommon, posing significant diagnostic and therapeutic challenges. We present a case of a 30-year-old woman at the 12th gestational week with fever, arthralgia, rash, cervical lymphadenopathy, cytopenia, and elevated liver enzyme. Bone marrow biopsy revealing hemophagocytosis, elevated ferritin and triglycerides, high interleukin-2, fever and cytopenia, confirmed the diagnosis of HLH. Further evaluation revealed the diagnosis of SLE. Treatment was initiated with intravenous immunoglobulin and corticosteroids. Given the deterioration in the patient’s clinical condition, a decision was made to terminate the pregnancy. She continued in the following months to receive SLE treatment with corticosteroids, cyclophosphamide, hydroxychloroquine, and later with mycophenolate mofetil due to the development of Class IV of lupus nephritis. P19V IgM antibodies were initially positive, later seroconverted to IgG, indicating that infection may have acted as a trigger for the onset of SLE and MAS development during pregnancy. The overlapping clinical features of P19V infection, SLE, and MAS pose significant diagnostic and therapeutic challenges. Early recognition and comprehensive diagnostic evaluation are crucial for the management of these conditions, especially during pregnancy, where both maternal outcomes are at risk.

## 1. Introduction

Hemophagocytic lymphohistiocytosis (HLH) and macrophage activation syndrome (MAS) are rare life-threatening hyperinflammatory conditions characterized by the excessive activation and proliferation of macrophages and T-lymphocytes, leading to cytokine release and hemophagocytosis. Traditionally, HLH can be divided into primary, due to inborn errors of immunity, and secondary, which is associated with various conditions, such as systemic autoimmune rheumatic disease, lymphomas, and infections. MAS is considered a form of secondary HLH, typically occurring in the context of autoimmune rheumatic diseases. However, due to the wide range of associated conditions, HLH is now recognized as a heterogeneous group comprising eight distinct categories: familial HLH; autoimmune inflammatory rheumatic diseases (AIIRD)/MAS; HLH induced by excessive activation of the inflammasome; HLH with immune impairment (primary immunodeficiencies or immune suppression-related); HLH occurring after immune activation therapies (e.g., Chimeric Antigen Receptor T-Cell Immunotherapy (CAR-T), cell therapy); HLH associated with malignancies (e.g., lymphoma); HLH induced by infections (e.g., Epstein–Barr Virus—EBV, Cytomegalovirus—CMV); and HLH without any identified trigger [1]. The 2022 EULAR/American College of Rheumatology guidelines use the term HLH/MAS as a syndrome with specific clinical and laboratory manifestations, that can be associated with genetic causes, predisposing conditions (juvenile idiopathic arthritis, systemic lupus erythematosus (SLE), lymphomas, certain metabolic conditions) that increase susceptibility and acute triggers (infections, immunotherapies) [2]. Although MAS can complicate various autoimmune diseases, its occurrence during pregnancy is exceptionally rare [3].

Parvovirus B19 (P19V) infection has been implicated in the pathogenesis of autoimmune disorders, including SLE [4]. It is hypothesized to act as a potential trigger for disease onset or exacerbation. P19V is identified by the presence of virus-specific IgM antibodies and/or viral DNA in peripheral blood, typically detected via quantitative PCR (qPCR). However, in some clinical settings where PCR testing is not available, serologic conversion from IgM to IgG may serve as a surrogate marker of recent infection. The virus exhibits tropism for erythroid progenitor cells but can also infect other cell types, not typically permissive to viral replication. This leads to a spectrum of hematologic and immunologic abnormalities. The proposed mechanisms underlying its autoimmune effects include molecular mimicry and virus-induced apoptosis, resulting in the presentation of self-antigens and subsequent T-cell activation [5,6]. P19V infection has been associated with both SLE and HLH/MAS [7,8]. P19V infection, MAS, and SLE share overlapping clinical manifestations, including fever, arthritis, cytopenias, elevated inflammatory markers, coagulopathy, hepatic dysfunction, lymphadenopathy, and organomegaly [9,10]. The overlap in both clinical features and potential etiological links among these three conditions can present considerable diagnostic and therapeutic challenges, particularly during pregnancy, when both maternal and fetal health are at stake. We present a case of MAS as the initial presentation of SLE in a pregnant woman, associated with P19V infection.

## 2. Case Report

A 30-year-old woman became pregnant with twins in July 2024. Several weeks into the pregnancy, she experienced a loss of appetite and nausea without vomiting, symptoms attributed to early gestation. The patient had no history of chronic illnesses, nor any family history of autoimmune diseases. She was under regular obstetric follow-up. In September 2024, she began experiencing generalized weakness and malaise, along with a fever up to 38.5 °C, sore throat, dysphagia, ear pain, arthralgia, cervical lymphadenopathy, and chest rash. Empirical treatment with an antibiotic was initiated, and she was admitted to the local general hospital. However, as her condition failed to improve by the 12th gestational week, she was referred to the tertiary Gynecology and Obstetrics Clinic. An ultrasound confirmed a viable twin pregnancy. On admission, the patient’s body temperature was 36.9 °C, she appeared pale, with unilateral cervical lymphadenopathy and mild bilateral lower limb pitting edema. The remainder of the physical examination was unremarkable. Laboratory investigations revealed normocytic anemia (Hb 92 g/L, MCV 81.8 fL), leukopenia (2.9 × 10^9^/L) with marked lymphopenia (Ly 0.4 × 10^9^/L), elevated C-reactive protein (CRP 51.7 mg/L; n.v 0–5 mg/L) and ferritin (1262 µg/mL; n.v 22–275 µg/mL). Liver function tests revealed elevated levels of aspartate aminotransferase (AST) 154 U/L; n.v 0–37 U/L), alanine aminotransferase (ALT) (85 U/L; n.v 0–41 U/L), alkaline phosphatase (ALP) (342 U/L; n.v 40–120 U/L), gamma-glutamyl transpeptidase (GGT) (277 IU/L; n.v 0–38 U/L). Abdominal ultrasound showed parailiac lymphadenopathy (up to 27 × 15 mm) without hepatosplenomegaly. A markedly elevated D-dimer level (2.69 g/L; n.v < 0.5 g/L) prompted echocardiography, which did not suggest pulmonary embolism. Low-molecular-weight heparin (LMWH) was initiated prophylactically. An ENT examination revealed pharyngeal hyperemia and enlarged tonsils. An infectious disease specialist recommended extensive serological testing, including screening for hepatitis B and C, HIV, herpes simplex virus, varicella-zoster virus, CMV, *Treponema pallidum*, *Toxoplasma gondii*, *Leishmania infantum*, *hantavirus*, *Brucella* spp., *rubella*, *Bartonella henselae*, *Francisella tularensis*, *Rickettsia* spp., *Ehrlichia* spp., *Borrelia burgdorferi*, *Coxiella burnetii*, PV19, and *Leptospira interrogans*. All tests returned negative results except for significantly elevated IgM for PVB19 and *Leptospira* spp. A reference microscopic agglutination test (MAT) for Leptospira was negative, ruling out leptospirosis. Empirical broad-spectrum antimicrobial therapy was initiated, along with LMWH, and supportive care.

First-trimester combined biochemical and ultrasound screening revealed high suspicion of trisomy 21 in both fetuses. On the fifth day of hospitalization, her condition deteriorated with the onset of tachycardia and decreased oxygen saturation, and worsening pancytopenia. The decision to terminate the pregnancy was made jointly with the patient, based on maternal clinical deterioration, high-risk fetal screening, ongoing systemic inflammation and poor response to initial treatment. This intervention was pursued to stabilize the mother’s health, prevent complication and facilitate the initiation of intensive immunosuppressive therapy. Bone marrow aspiration revealed single macrophages with cellular debris, hemophagocytes, and mild macrophage activation. Elevated levels of soluble IL-2 receptor (s IL-2R 957 U/mL; n.v < 350 U/mL), and triglycerides (2.11 mmol/L; n.v 0–1.7 mmol/L) were found. The diagnosis of HLH was made based on fever, pancytopenia, elevated ferritin and triglycerides, hemophagocytes in the bone marrow, elevated sIL-2R, resulting in a HScore of 195. Treatment was initiated with intravenous methylprednisolone (1 g/day for 3 days), followed by intravenous immunoglobulin (400 mg/kg/day for 2 days) (Timetable)

Immunological testing was obtained and notable for antinuclear antibodies (ANA) on HEp-2 cells were positive at >1:640, showing a nucleoplasmic and mitotic apparatus homogeneous pattern. Additional autoantibodies were also positive, including anti-double-stranded DNA (1:320), anti-histone antibodies (>200 U/mL; n.v: <20 U/mL), anti-nucleosome antibodies (>200 U/mL; n.v: <20 U/mL), anti-Sm/RNP, extractable nuclear antigen screen (75.9 RU/mL; n.v: <20 RU/mL), anti-beta-2-glycoprotein I IgM (82.8 U/mL; n.v: <50 U/mL), and anti-cardiolipin IgM and IgG (25.5 U/mL and 25.9 U/mL, respectively; n.v. for both: <12 U/mL). Complement levels were reduced C3 0.25 g/L (n.v: 0.8–1.85 g/L) and C4 0.02 g/L (n.v: 0.1–0.4 g/L). The total serum IgG 12.3 g/L (reference range 5.5–16.3 g/L) and IgM 1.4 g/L (reference range 0.3–2.9 g/L) concentrations were within normal limits. The immunologist was consulted, and the patient was diagnosed with SLE, based on the following EULAR classification criteria: fever, arthralgia, leucopenia, positive ANA HEp2, positive anti-dsDNA and anti-Sm antibodies, proteinuria, low C3 and C4 complement, and positive antiphospholipid antibodies. SLE Disease Activity Index 2000 (SLEDAI-2K) score was 21. Based on the overall findings, a diagnosis of SLE was established, with MAS as the predominant clinical manifestation.

**Figure 1:**
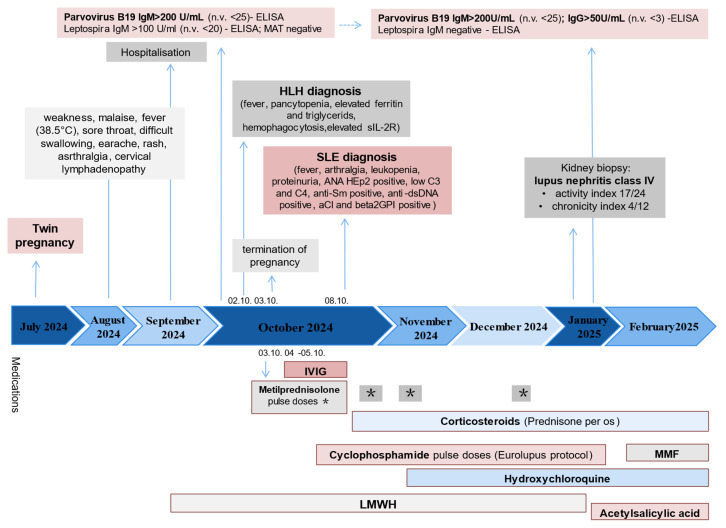
Timeline of the case report. Abbreviations: MAT—microscopic agglutination test, HLH—hemophagocytic lymphohistiocytosis, IL2R—interleukin 2 receptor, SLE—systemic lupus erythematosus, ANA—antinuclear antibodies, aCl—anticardiolipin antibodies, IVIG—intravenous immunoglobulins, mycophenolate mofetil, LMWH—low-molecular-weight heparin, * methylprednisolone pulse doses.

The patient was transferred to the Clinic for Allergy and Immunology. Upon admission, she had persistent lymphopenia (Ly 0.3 × 10^9^/L), significant anemia (Hb 69 g/L) requiring a blood transfusion, and a nephrotic-range proteinuria (5.27 g/24 h). Inflammatory markers were elevated (ESR 83 mm/h, CRP 43.5 mg/L, ferritin 1295 ng/mL). Hepatic enzymes normalized during treatment, except for persistently elevated GGT (102 IU/L). Magnetic resonance imaging (MRI) revealed gallbladder microlithiasis without signs of acute cholecystitis, and magnetic resonance cholangiopancreatography (MRCP) showed normal biliary anatomy. Pulse methylprednisolone therapy (1 g/day for 2 days) was followed by tapering corticosteroids, hydroxychloroquine (200 mg), and cyclophosphamide (CYC) 500 mg biweekly in accordance with the Euro-Lupus protocol. The patient initially declined a kidney biopsy, and CYC therapy was continued. Following the sixth CYC dose (total 3 g), anemia persisted (Hb 114 g/L), with ongoing hematuria, pyuria, and proteinuria (4.47 g/24 h), a kidney biopsy was eventually performed after the seventh CYC dose (3.5 g total), which confirmed diffuse proliferative lupus nephritis, Class IV A/C (activity index 17/24, chronicity index 4/12). Treatment was switched to mycophenolate mofetil (2 g/day), leading to clinical and laboratory improvement. Acetylsalicylic acid was added to her regimen as prophylaxis against thrombosis, given positive anticardiolipin and anti-β2-glycoprotein antibodies, as well as livedo reticularis. C3 and C4 concentrations remained persistently low throughout treatment and did not normalize. Although acquired complement consumption is a hallmark of an active SLE, the persistent hypocomplementemia observed in our patient, despite immunosuppressive therapy, raises the possibility of an underlying congenital deficiency. The coexistence of both mechanisms cannot be excluded and may have contributed to disease severity. Serial P19V serologies confirmed persistent high IgM titres and subsequent IgG seroconversion, indicating association of P19V with SEL/MAS development (Figure 1, Table 1).

## 3. Discussion

MAS is a rare, potentially fatal hyperinflammatory complication associated with various systemic autoimmune rheumatic diseases, including SLE. The estimated prevalence of MAS among SLE patients ranges from 0.9% to 4.6%, with reported mortality rates between 5% and 35% [11,12]. Due to the clinical complexity and heterogeneity of both SLE and MAS, distinguishing between the two conditions can be challenging. However, MAS and SLE are not mutually exclusive entities, and MAS may occur at any stage during the course of SLE. Rarely, MAS can represent the initial clinical manifestation of SLE, with case reports documented in the literature [13,14,15,16]. In a retrospective analysis of 86 patients with SLE complicated by MAS, 47 (54.65%) were found to have MAS as the initial presentation of SLE [17]. SLE patients presenting as MAS had significantly higher SLEDAI-2K scores, lower complement C3 and C4 levels, and a higher frequency of oral ulcers and serositis compared to those who developed MAS during the established course of SLE. The authors concluded that high SLE disease activity may be a trigger for MAS onset.

In the case we present, MAS developed as the initial manifestation of SLE in the 12th week of pregnancy. To the best of our knowledge, only eight such cases have been reported in the literature, with a median maternal age of 27 years (range: 20–31), occurring in the first or second trimester [18]. Of the seven cases reviewed, one maternal death was reported, and five pregnancies resulted in abortion or fetal demise. Our patient initially presented with fever, lymphadenopathy, cytopenia, and elevated liver enzymes, features that prompted a broad differential diagnosis, including infection, HLH, hematological malignancies, HUS (Hemolytic Uremic Syndrome)/HELLP Syndrome, and autoimmune diseases. Elevated ferritin levels, along with bone marrow biopsy findings, directed further evaluation towards the diagnosis of HLH, meeting the established diagnostic criteria. Subsequently, the detection of multiple SLE-associated autoantibodies confirmed the diagnosis of SLE.

Diagnosing MAS in the context of new-onset SLE during pregnancy is particularly challenging due to the overlapping, nonspecific features of both conditions. No specific diagnostic criteria currently exist for MAS in SLE in pregnant patients. In clinical practice, diagnosis is often made by HLH-2004 criteria and using HScore [19,20,21]. According to the HLH-2004 criteria, fulfillment of five out of eight criteria is sufficient for diagnosis [19]. Notably, the absence of a single criterion, such as hemophagocytes, does not exclude the diagnosis. The HScore, which incorporates additional parameters such as immunosuppression and AST levels, estimates the probability of secondary HLH. A score above 169 is considered indicative of a high probability of secondary HLH, with a sensitivity of approximately 90% [20]. In the review of SLE/MAS cases during pregnancy, only four demonstrated hemophagocytic features on biopsy [18]. Biopsy may not always confirm hemophagocytes due to the disease’s evolving nature. It can occur outside the bone marrow, so repeated biopsies or use of the HScore may be needed when clinical suspicion remains high. Ferritin has emerged as a key biomarker in the early diagnosis of MAS, even in cases where it serves as the presenting feature of SLE [22,23]. Markedly elevated ferritin levels, in conjunction with supportive biopsy findings, prompted further evaluation for a diagnosis of HLH in our patient. She met the HLH-2004 diagnostic criteria and had an HScore of 169, indicating a high probability of HLH. However, in pregnant women, with subtle or mild early symptoms, reliance solely on the HLH-2004 criteria or on a single biopsy may delay diagnosis. Thus, the diagnosis of HLH/MAS in the context of newly diagnosed SLE in pregnancy requires a comprehensive approach, integrating clinical evaluation with multiple diagnostic tools.

Identifying the underlying etiology of MAS is often challenging, and this difficulty is amplified if both SLE and MAS are present in pregnancy, as is our patient’s case. Due to the low incidence of SLE-associated MAS in pregnancy, most of the literature focuses on HLH in pregnancy, which includes autoimmune-related cases. In a review of 81 reported cases of HLH during pregnancy, infections were identified as the leading etiologic factor, accounting for 41% of cases [24]. Other causes included malignancies (3/81) and genetic factors (1/81). Among infectious agents, EBV was the most commonly identified, followed by herpes simplex virus, P19V, CMV, *Leishmania donovani*, varicella-zoster virus, malaria, *Mycobacterium tuberculosis*, adenovirus type 7, hepatitis B surface antigen, *Staphylococcus epidermidis*, and *Acinetobacter haemolyticus*. In our patient, an extensive microbiological evaluation was performed, given the initial presentation with a fever, rash, cervical lymphadenopathy, elevated CRP, and cytopenias, all of which could be attributed to an infectious etiology. Initial serologic testing revealed IgM positivity for Leptospirosis; however, MAS-specific testing and clinical evaluation ruled it out. A possible explanation for the false-positive IgM result includes polyclonal B-cell activation or cross-reactivity with IgM anti-β2GPI or IgM anticardiolipin antibodies.

Although the association between HLH and P19V is rare, it has been documented in several case reports [25,26,27], including in pregnancy settings [28,29]. Furthermore, clinical and laboratory overlap between P19V infection and SLE has long suggested a potential pathogenetic relationship [7,30,31]. Common features, such as anemia, arthralgia, fever, malaise, lymphadenopathy, rash, and overlapping autoantibody profiles, can lead to diagnostic confusion [7]. Our patient initially presented with fever, malaise, rash, and arthralgia, symptoms that may be attributed to either SLE or acute P19V infection. The association between P19V and SLE has been described in three clinical scenarios: (1) P19V infection may mimic SLE; (2) it may exacerbate existing SLE; or (3) it may trigger new-onset SLE in previously healthy individuals [32].

P19V is a single-stranded DNA virus of the *Erythrovirus genus*, with a marked tropism for erythroid progenitor cells in the bone marrow. Parvovirus B19 can be transmitted via multiple routes, including respiratory droplets, vertical (mother-to-fetus) transmission, and transfusion of infected blood products. Given that our patient developed clinical manifestations prior to receiving any blood transfusions, respiratory droplet transmission is considered the most likely source of infection in this case. While P19V infection is typically mild and self-limiting, it can be severe in immunocompromised hosts [33]. The diagnosis of recent or active P19B infection relies on the detection of virus-specific IgM antibodies and/or viral DNA in peripheral blood, typically via quantitative PCR (qPCR) assays [34]. Our patient initially received IVIG, which may have contributed to a false-positive P19V IgG result, due to passive antibody transfer from donor plasma. However, the observed seroconversion, with IgG detected more than four months after IVIG administration, supports the true P19V infection rather than IVIG-induced seropositivity.

**Figure 2:**
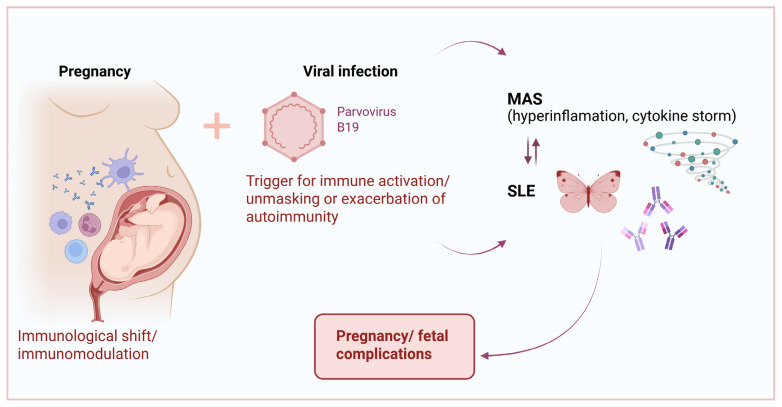
The complex interactions between P19V, MAS, and SLE in pregnancy.

While both SLE disease activity and infections are recognized triggers of MAS, the predominant pathophysiological mechanism in pregnancy-associated SLE/MAS remains unclear [3,8]. HighSLE activity in our patients may have contributed to the severe clinical course and pregnancy loss. Acute P19V infection is associated with immune-cell activation and cytokine dysregulation. Specifically, lymphocytes respond to the viral capsid proteins (VP1 and VP2) by producing IL-2 and IFN-γ, while the non-structural protein NS1 promotes transcription of IL-6 [35,36]. In susceptible individuals, particularly those with underlying autoimmune predisposition, P19V infection may act as a trigger for SLE by promoting the presentation of self-antigens and enhancing T-cell activation. Concurrently, excessive cytokine production—dominated by IFN-γ, IL-6, IL-10, and TNF-α—can promote macrophage activation and cytotoxic T-cell proliferation, leading to the cytokine storm characteristic of MAS. On the other hand, HLH arises from the uncontrolled proliferation and activation of CD8 cytotoxic T cells and natural killer cells, leading to the excessive release of pro-inflammatory cytokines, such as tumor necrosis factor-α (TNF-α), and IL-1, IL-4, IL-6, IL-8, IL-10, IL-18 commonly referred to as a cytokine storm [37]. This cytokine storm, superimposed on the immune dysregulation due to SLE and the altered immunologic environment of pregnancy, may have contributed to the life-threatening presentation, ultimately leading to pregnancy loss (Figure 2).

Therapeutic management of SLE and MAS in pregnancy is particularly challenging due to the potential risks to the fetus. There is currently no established consensus on the diagnosis and treatment of HLH or MAS during pregnancy, necessitating individualized and multidisciplinary approaches. The treatment of MAS associated with autoimmune and immune-related diseases, such as SLE, is guided by clinical experience and extrapolation from therapeutic protocols used in other forms of HLH [24,38]. The primary goals are to suppress the life-threatening hyperinflammatory response and to address the underlying etiological triggers. High-dose corticosteroids, remain the cornerstone of initial therapy. In cases of inadequate response, immune suppressive and immunomodulatory agents such as cyclosporine, etoposide, IVIG, and therapeutic plasma exchange have all been employed with varying degrees of success [18,24,38,39].

New-onset SLE during pregnancy, high disease activity at presentation, and renal involvement are all recognized as risk factors for adverse fetal outcomes [40,41]. These factors, in combination with MAS and acute P19V infection, could have contributed to a severe clinical presentation that led to the decision to terminate the pregnancy. Termination of the pregnancy should also be considered if there is a lack of response to treatment for MAS during pregnancy, but this issue is very controversial [18]. Our approach aligns with current recommendations of the American College of Obstetricians and Gynecologists (ACOG), which support the individualized management of pregnancy in patients with severe maternal disease, prioritizing maternal health when continuation of pregnancy poses a significant risk [42]. Prompt recognition of the triad SEL/MAS/infection is very important, as delayed treatment can result in poor maternal and fetal outcomes.

## 4. Conclusions

The overlapping clinical and laboratory manifestations of P19V infection, SLE, and MAS, along with their potential etiological and causal relationship, may significantly complicate the diagnostic process during pregnancy. This case illustrates the complex interplay between pregnancy-related immunological changes, viral triggers, and autoimmunity, underscoring the critical need for the early recognition and coordinated, multidisciplinary management of MAS and SLE in pregnancy to ensure the best possible outcomes for both the mother and fetus.

## Figures and Tables

**Table 1:** Selected laboratory findings throughout different points in time.

Analysis		On Admission	Before CS Therapy	After 6th CYC Pulse	Reference Range
Complete blood count	WBC	2.9	2.8	4.3	3.4–9.7 × 10^9^/L
	th	2.3	2.2	2.5	2.1–6.5 × 10^9^/L
	Ly	0.4	0.05	1.2	1.2–3.4 × 10^9^/L
	RBC	3.26	3.22	3.95	3.86–5.08 × 10^12^/L
	Hb	92	82	114	119–157 g/L
	PLT	259	113	277	150–450 × 10^9^/L
Markers of inflammation	ESR	-	100	42	<10 mm/h
	CRP	51.7	55.5	10.3	<5 mg/L
	Ferritin	1262.3	1648.3	163.5	4.6–204 µg/L
Liver function	AST	145	189	47	<37 U/L
	ALT	85	91	74	<41 U/L
	GGT	277	255	113	<38 U/L
	ALP	342	-	98	40–120 U/L
	TGs	-	2.11	1.92	0–1.7 mmol/L
Kidney function	Urea	1.9	2.1	4.7	2.5–7.5 mmol/L
	Creatinine	40	41	60	45–84 umol/L
	eGFR	>60	>60	>60	>60 mL/min/ 1.73 m^2^
	Urine sediment	-	Erythrocytes leucocytes	Proteins, hemoglobin erythrocyte leucocytes	-
	24 h-proteiuria (g/24 h)	-	2.03	3.83	<0.5 g/day

Abbreviations: CS—corticosteroid; CYC—cyclophosphamide; WBC—white blood cells; Ne—neutrophils; Ly—lymphocytes; RBC—red blood cells; Hb—hemoglobin; PLT—platelets; ESR—erythrocyte sedimentation rate; CRP—C-reactive protein; AST—aspartate transaminase; ALT—alanine transaminase; GGT—gamma-glutamyl transferase; ALP—alkaline phosphatase; Chol—cholesterol; TGs—triglycerides; eGFR—estimated glomerular filtration rate.

## Data Availability

Data are contained within the article.
